# Experimental Study on the Corrosion of Carbon Steel and Aluminum Alloy in Firefighting Protein Foam Concentrates

**DOI:** 10.3390/ma14237259

**Published:** 2021-11-27

**Authors:** Marina Teodora Patrascu, Andrei Dan Busuioc, Cristina Busuioc, Adina Cotarta, Anca Cojocaru, Teodor Visan, Danut Ionel Vaireanu

**Affiliations:** 1 Faculty of Applied Chemistry and Materials Science, University Politehnica of Bucharest, 060042 Bucharest, Romania; marina.patrascu@ymail.com (M.T.P.); jinga_cristina@yahoo.co.uk (C.B.); adina@cael.pub.ro (A.C.); t_visan@chim.upb.ro (T.V.); di_vaireanu@yahoo.co.uk (D.I.V.); 2 General Inspectorate for Emergency Situations (IGSU), 023765 Bucharest, Romania; busu1569@gmail.com

**Keywords:** corrosion of fire fighting equipment, protein foam concentrate, carbon steel, AlSiCuMg aluminum alloy, methods for corrosion rate determination

## Abstract

The corrosion of mild steel and Al alloy in Fomtec P 6% and 6% P Profoam 806 protein-based foam concentrates was investigated. Weight-loss data for steel showed corrosion penetration of 0.745 mipy in Fomtec and 2.269 mipy in Profoam, whereas for Al alloy the penetration levels were 0.474 and 1.093 mipy, respectively. Scanning electron microscopy and energy dispersive X-ray spectroscopy allowed characterization of the metallic surface covered or free from corrosion products. Values of corrosion potential, corrosion current density and corrosion penetration were calculated by using potentiodynamic polarization curves. Electrochemical impedance spectra illustrated the change in polarization resistance during anodic polarization. Data obtained by accelerated electrochemical methods confirm the greater aggressiveness of the Profoam concentrate compared to Fomtec concentrate.

## 1. Introduction

Corrosion of metallic materials is one of the most significant issues for all industrial sectors, among which are sewage pipelines and firefighting service, involved in terms of both lifecycle cost and system reliability [1]. It has been shown that corrosion problems, when working with protein-based agents, can occur on a wide range of complex devices, especially for storage and proportionating systems.

Corrosion processes occur in foam storage tanks, piping, in-line inductors (eductors) such as Venturi tubes, hose couplings and nozzles—all being exposed to firefighting agents used as either liquid concentrates or as already prepared foam [2,3,4,5]. Corrosion is known to produce many problems in fire protection systems, especially wet and preaction fire sprinklers [6,7,8,9,10,11,12,13,14]. Carpen et al. [15,16] analyzed the failure of stainless steel pipes in fire protection systems of a hot rolling mill and of a power plant, in contact to natural or tap water containing chloride, manganese and iron ions. Su and coworkers [4,17,18,19,20] showed that corrosion products such as tubercles and scale are deposited on galvanized and carbon steel, impairing the effectiveness of sprinkler systems.

The last two decades have seen a number of advances in the quality of firefighting foams [21,22,23,24,25,26] capable of rapidly suppressing and extinguishing the class B pool fires. Aqueous film forming foams (AFFFs) have gained much attention because they are the most effective fire extinguishing agents for fighting hydrocarbon liquid fuel fires and chemical solvent fires. Today, all available AFFF concentrates have in their chemical compositions a fluorocarbon surfactant (as a key component), a hydrocarbon surfactant and an organic solvent [27]. In practice, the foam is routinely prepared by diluting the commercial 1–6% extinguishing concentrate with water and forcing the gas (air, nitrogen) into this solution. Unfortunately, it was noticed that the fluorinated (or polyfluorinated) surfactants possess complex environmentally harmful behavior because they are persistent, potentially bio-accumulative and toxic.

In order to decrease the toxicity and cost, regular protein-based and fluoroprotein foams were widely introduced in practice and are expected to have good biodegradability [28,29,30]. The protein-based concentrates are fabricated from hydrolyzed protein obtained from either animal keratin or a vegetable source; a suitable solvent; a stabilizer; and a preservative. The added fluorinated surfactant makes them more effective. Concentrates for film forming fluoroprotein foam and corresponding alcohol resistant aqueous film-forming foam are more interesting due to their better firefighting performance, better burn-back resistance and lack of degradation over time.

However, the use of conventional aqueous film-forming foam is now severely restricted, and AFFFs must be replaced without sacrificing fire suppression performances. Notably, a group from the US Naval Research Laboratory [31,32,33,34,35,36] has recently showed that a difficulty with replacing fluorocarbon surfactants in AFFFs is due to the complexity of commercial formulations; another problem is that the effects of individual component on foam properties are not well understood. An ecological alternative would be the use of synthetic concentrates for fluorine-free foams [37,38,39,40], but it seems that these proposed solutions cannot yet extinguish as efficiently as AFFFs [31,41]. Protein-based formulations were identified as alternative solutions/replacements. Currently, protein-base foams are marketed by Profoam Corporation, Rutledge, Georgia, US (Provex AR 6-6), Angus Chemical Company, Buffalo Grove, Illinois, US (perfluoroalkyl substance-based foams TF3 and TF90) and Dr. Sthamer company GhmbH & Co., Hamburg, Germany (Foamousse). In general, fluorine-free foams exhibit much better foam stability but worse foam spread property than commercial AFFF. Only a fluorine-free foam containing a silicone surfactant showed better fire extinguishing and burn-back performance [40].

In firefighting systems, the walls of large storage vessels, commonly made of low carbon steel (cold-rolled), get corroded due to the foam concentrate that may drill the metal. Corrosion has caused problems over the years, and alternative materials had to be considered. Repair welding is expensive, and other options are used; for instance, storage tanks could be fabricated of carbon steel covered with baked phenolic lining and certain room temperature-cured coatings [42]. Carbon steels A53–A106 are not recommended for storage of protein-based AFFFs due to the corrosiveness of protein foam agents [43]. To overcome these issues, carbon steel was gradually replaced, and 304 or 316 austenitic stainless steel started to be used. Additionally, the foam concentrate piping was recommended to be made of stainless steel and brass, whereas cast iron and bronze remained the materials for the construction of pumps and valves.

The nozzles made from aluminum alloys are damaged by the corrosive attack of the foam during repeated usage. In Romania, firefighters are using Storz type couplings (hose couplings, adapters and blank caps) fabricated of die cast aluminum alloy. Unfortunately, the most used metallic materials in their fire service’s technical arsenal tend to be more susceptible to localized corrosion, which is often difficult to detect and to monitor effectively. Moreover, Al alloys may take up hydrogen during their processing and use, and the absorbed hydrogen is transported into the alloy and may be trapped at different preferred sites. Some researchers have reported, very recently, that during the formation of the protective oxide layer on the surface of commercial aluminum alloys, the crack growth is enhanced [44,45] due to absorption of reactive atomic hydrogen produced by the humidity from the environment. The electrochemical process is: H_2_O + e^−^ → OH^−^ + H_abs_. One consequence is that crack propagation around second-phase particles or grain boundaries can be ten times worse in humid air than in a vacuum, leading to hydrogen embrittlement susceptibility of the aluminum alloys. Thus, the question of corrosion of metallic construction materials due to fire extinguishing agents has become an issue of concern, and one of the most difficult problems in the fire service.

In fire engineering, the corrosion of carbon steel and aluminum alloys in contact with firefighting foams used to effectively extinguish fires has not yet extensively been studied. One of the first studies in the United States of the corrosion characteristics of a protein-type foam was conducted on cold-rolled carbon steel, 304 stainless steel and 1100 and 5052 aluminum alloys [46]. Stainless steel has shown an excellent corrosion resistance compared to carbon steel. The research involving the aggressiveness of a protein-based agent in comparison with an AFFF (FC-195 light water) has been continued with the above-mentioned steels and other common metals and alloys (Cu, Pb, Zn, Ti, brass, Monel and CuNi alloy) [2]. The corrosivity of the standard protein concentrate toward carbon steel has been found to be about 13 times higher than of light water, whereas stainless steel had a corrosion rate of zero. The corrosion properties of seven foam firefighting agents (among them were 3M FC 203 CE, Angus FP 70 and National Foam XL3, all with 21–38 g/L Cl^−^ content) were examined by Scheffey and Wright [3]. By testing common construction materials, carbon steel (UNS G10100), 304 stainless steel and 6061 aluminum alloy, the authors found that the corrosion rate was not proportional to the halide content of the extinguishing agent. Angus FP 70 was found to be the most corrosive for carbon steel. A study of metal compatibility with fluoroprotein surfactant and fluoropolymer-free foam concentrates (RF Solberg as 3%, 6% and 3 × 6 ATC formulations) was also performed [41]. Immersion testing was done for pure metals (Al, Cu), cast iron, mild and stainless steels and alloys (brass, bronze, CuNi, Monel, alloys of Mg or Ti). The weight losses in the coupons determined in comparison with values in distilled water showed that water is more aggressive than concentrates or their diluted solutions.

Few corrosion studies involving a firefighting service have been performed outside the United States. Papers published by Radwan et al. and Rakowska et al. [5,47,48] described uniform and localized corrosion of fire extinguishing equipment, suggesting the most important methods of corrosion prevention or mitigation. Kostyaev et al. [49] have investigated the corrosiveness of PO-6TS-M concentrate (AFFF from IvKhimProm AO, Ivanovo, Russia), which comprises the PO-6TS fluorochemical surfactant and ethylene glycol. The corrosion behavior of St3 carbon steel in a premixed aqueous solution (concentrate diluted in a 1:17 ratio) was evidenced by measuring weight loss and by potentiodynamic polarization. The introduction of more than 0.5 g/L additives from antipyrene class ensured complete steel passivation. Elahresh and Jewilli [50] evidenced the corrosion on 304 stainless steel in 3% Hydral foam concentrate (from Sabo Foam SRL, Bergamo, Italy), which is a highly efficient type of synthetic AFFF fire suppressant agent. Experiments showed close agreement between weight loss data and electrochemical measurement (anodic polarization).

The main purpose of the present research was to study the corrosion of carbon steel and an aluminum alloy, which are commonly used as construction metallic materials in firefighting, in Fomtec P 6% and 6% P Profoam 806 aggressive foam concentrates. To the best of our knowledge, this is the first study about the corrosivity of these commercial protein-based foam concentrates.

## 2. Materials and Methods

The investigated metal corrosion behavior refers to equipment in fire extinguishing vehicles, e.g., Iveco, Volvo, Dac-Lancer, APCA (including APCA-T and APCAA R 12215 DFA) trucks used by Romanian firefighters. Steel samples were taken from 4 mm thick walls of the containers and pipes made from cold-rolled low carbon steel. This low cost mild steel is named in Romania as OL 42.2 k steel (SR EN 10025-A1/1994) and is similar to S275 JR steel (EN 10025-2/2004), RSt 42.2 steel (DIN 17100), Fe 430B steel (ISO 630), or 50 grade steel (ASTM A529). It has nominal composition (wt%): 0.6 Mn; 0.33 C; 0.045 P; 0.045 S; Fe-balance. Selected Storz hose couplings samples were made from cast aluminum alloy with a composition closer to AlSi5Cu1Mg alloy (EN AC-45300), with a content (wt%) in limits of: 86–93 Al; 5–13 Si; 1.0–1.5 Cu; 0.6–0.75 Mg; 0.2 Fe; 0.2 Ti; 0.2 Ni; 0.1 Mn; 0.1 Zn.

The investigated fluorine free foam concentrates considered as the aggressive media were Fomtec P 6% (Dafo Fomtec AB, Stockholm, Sweden) and 6% P Profoam 806 (Profoam, Profoam International, Paris, France) commonly used by Romanian firefighters. Due to its proprietary nature, the chemical composition of foam concentrates is not generally published by the manufacturers. We only know that both protein foam concentrates are produced by a controlled blend of hydrolyzed animal proteins (70–0 wt%), hexylene glycol (<6 wt%), diclorometaxylenol (<0.5 wt% as bactericide), and foam boosters, antifreeze agents, stabilizers and preservatives. The concentrates also contain 8–15 wt% metal salts as chlorides (sodium, manganese) and <0.2 wt% sulphates (iron). The pH of concentrates, measured by us, was in the range of 9 to 10, which is slightly more alkaline than the values given by the manufacturer (pH 6–8). Both dark brownish liquids are non-toxic and biodegradable and should be diluted with fresh or sea water at a 6-part concentrate to 94-part water ratio.

For weight-loss determination the steel specimens were cut into rectangular strips with 25–28 cm^2^ surface area. Large curved or even profiled coupons of Al alloy having 15–17 cm^2^ surface area were cut from Storz hose couplings. The surface was finished by polishing it successively with SiC emery paper up to 2500 grit, then rinsed successively with tap water, deionized water, cleaned with acetone, dried with flowing air and finally weighed.

The measurements were conducted at ambient temperature (22 ± 2 °C) under vertical immersion of prepared metal specimens into beakers filled with equal volumes of 150 mL foam concentrate (aerated and refreshed periodically). At the end of gravimetric test some coupons were gentle scrubbed, rinsed with deionised water, allowed to dry in laboratory air and analyzed by microscopy. The corrosion products were removed from surface by brushing, intense rinsing with deionized water, drying and then each coupon was re-weighed accurately. Weight loss was determined with an analytical balance with a precision of 0.1 mg at different time intervals, the longest exposure period being 348 days for steel and 14 days for Al alloy.

The surface morphology of the exposed samples with and without corrosion products was observed by scanning electron microscopy (SEM) using FEI Quanta Inspect F (Philips, Eindhoven, Netherlands) microscope equipped with energy dispersive X-rays (EDS) device ((FEI Company, Hillsboro, OR, USA).) for elemental microanalysis. Both Everhart-Thornley secondary electron detector (ETD) (FEI Company, Hillsboro, OR, USA). and circular backscatter detector (CBS) (FEI Company, Hillsboro, OR, USA). were used for obtaining SEM images.

Laboratory-accelerated corrosion experiments as electrochemical testing were performed using a SP-150 BioLogic Sci. Instr. Potentiostat (BioLogic, Seyssinet-Pariset, France). All sample materials and foam concentrates used in these tests were identical to those used in the weight loss measurements. In the electrochemical cell the working electrode had a 1 cm^2^ exposed surface area of either 42.2 k steel or cast Al alloy by embedding in epoxy resin/polytetrafluoroethylene composite. A platinum plate (10 cm^2^) and Ag/AgCl/3 M KCl electrode ( Metrohm AG, Herisau, Switzerland were the auxiliary electrode and reference electrode, respectively. Semi-logarithmic potentiodynamic polarization curves were recorded with 3 mV/s sweep rate around the open-circuit potential to study the corrosion behavior of metallic samples. The specimens were immersed 30 min in the foam concentrate prior to the measurement in order to stabilize the open-circuit potential. Values of corrosion potential and corrosion current density were calculated at the intercept of the anodic and cathodic Tafel lines. The polarization resistance (a parameter which is inversely proportional to corrosion current) was determined experimentally by using electrochemical impedance spectroscopy (EIS). EIS spectra as Nyquist and Bode diagrams were obtained by using a superimposed 10 mV peak-to-peak ac voltage perturbation for the scanning frequency range from 100 kHz to 50 mHz and by polarizing the sample surface at various electrode potentials. The impedance data were further processed using an impedance module (FRA) of potentiostat and were modeled with ZView 2.4 software from Scribner Association Inc., Southern Pines, NC, USA. All electrochemical tests were conducted in freshly foam liquid concentrates at 22 ± 2 °C, and the results were repeated to check the reproducibility.

## 3. Results and Discussion

Being derived from natural products, the used protein-based foam concentrates are very quickly biodegradable compared to alternatives, and they do not require the use of environmentally harmful glycol additives, which are necessary in synthetic-based foams, especially for low-temperature use. It would be unsurprising for these organic substances not to lead to intense corrosion. However, as we mentioned above, the investigated protein foam concentrates contain inorganic salts (chloride, sulphate), which can be more aggressive. This contribution describes the experimental program undertaken to test the corrosivity of Fomtec P 6% and 6% P Profoam 806 firefighting foam concentrates in storage steel vessels or flowing into piping and Storz couplings made of Al alloy (with composition closer to AlSi5Cu1Mg alloy) for fire hoses. Their effects on the corrosion of carbon steel and Al alloy have not been systematically reported until now.

### 3.1. Long Exposure Study by Weight Losses

The gravimetric method of weight loss determination is the most widely used method of corrosion assessment due to its simplicity and reliability. The long exposure time of coupons immersed in liquid foam concentrates is important, since quick tests of accelerated corrosion give results which can sometimes be misleading. Unfortunately, the extremely dark color and opacity of the concentrates precluded the visual observation of the presence of metal ions in solution, and also of other accompanying insoluble corrosion products. Experimentally determined weight loss was converted to either gravimetric corrosion index (kg), or expressed as milligrams per square decimeter per day (mdd) or penetration rate *CR* (milli-inch per year, mipy), meaning the average depth of metal loss. We considered 8760 h to constitute a year.

#### 3.1.1. Corrosion of Carbon Steel

Table 1 displays the determined corrosion parameters for 42.2 k steel after different exposure time periods of up to 348 days. In calculations of corrosion penetration, a 7.86 g/cm^3^ density value of 42.2 k steel was taken. Corrosion attack levels were measured as integral weight losses, but the data allowed us to calculate them incrementally too. We supposed a uniform penetration rate, which is an engineering guide for designing the life expectancy of wall for tanks or pipes. The data obtained were fairly reproducible.

The gravimetric experiments showed that corrosion rate of steel in Fomtec P 6% increased continuously over time. Steel samples in Profoam concentrate were much more corroded over the entire exposure period. However, the largest value of corrosion rate was after the first 81 days of immersion. Then it was clearly attenuated, suggesting a steady-state corrosive attack within the period of 81–232 days (see differential values denoted by *). It seems that a stationary state was kept for longer periods of time in both corrosive media. As we discuss below for SEM images, this can be explained by the behavior of the layers formed by continuous corrosion (uniform corrosion) of the Fe component of steel. The corrosion products formed by interaction with protein and Cl^−^ (and SO_4_^2−^) from the foam solution can remain as a porous layer or detach from the metal surface and dissolve via diffusion into the bulk solution, so this layer does not protect the rest.

Our data are in good agreement with values of corrosion rate determined previously. Bertschy et al. [2] reported that the storage of light water concentrate in a carbon steel vessel produced a corrosion rate of 18.2 mdd. Values of penetration rate of 0.6–1.4 mipy for rolled low carbon steel (UNS G 101100) in protein foam concentrate were determined by Scheffey and Wright [3], who mentioned that the 1.6 mipy value is the maximum permissible corrosion rate in the US for this steel in a foam, under MIL-F-24385C. Our results obtained for steel corrosion in Fomtec fall within this allowed limit, whereas values for Profoam slightly exceed this limit. However, our data are significantly lower than the values for cold-rolled carbon steel of 18.2 mdd determined in a light water concentrate and of 134 mdd in a very corrosive protein foam concentrate [46].

Prior corrosion experiments, SEM images and EDS elemental analysis of 42.2 k steel showed that it has a surface morphology and a chemical composition with good homogeneity and uniform distribution of iron, manganese and other constituents. Following exposure to foam concentrate, the steel samples were observed to have undergone visible degradation. In general, as the exposure time was prolonged, the color of sample became darker. Additionally, we noticed during weight-loss experiments a change in color in some surface locations from yellow-brown (phase assigned to β-FeOOH) to green blue-greyish (phase assigned to the oxides/hydroxides mixture of Fe(II)/Fe(III) as a stable film). Due to the expansive nature of the corrosion products, it is possible to develop mechanical stress in the corrosion layer, thereby inducing two opposing effects—namely, pore blocking and the formation of cracks. Besides uniform corrosion, the SEM images of steel samples showed pitting corrosion.

**Figure 1:**
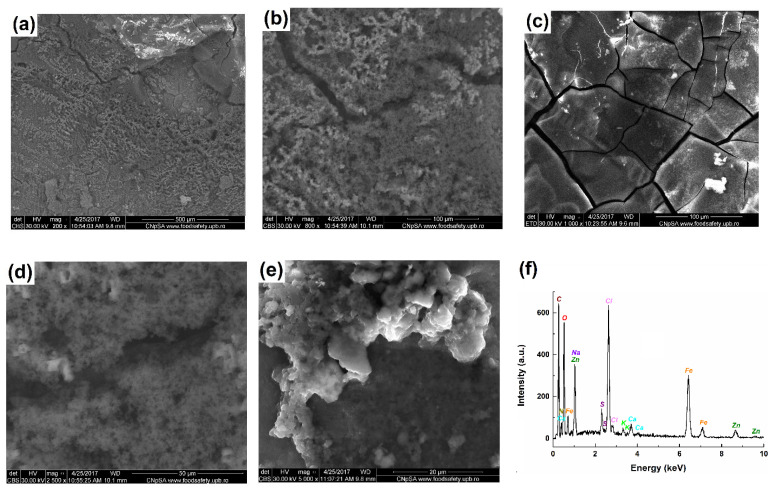
SEM micrographs of steel (sample 1) covered with corrosion products formed after exposure for 166 days in Fomtec (**a**–**e**); magnification from ×200 (**a**) to ×5000 (**e**); EDS spectrum showing elemental composition of corrosion products (**f**).

**Figure 2:**
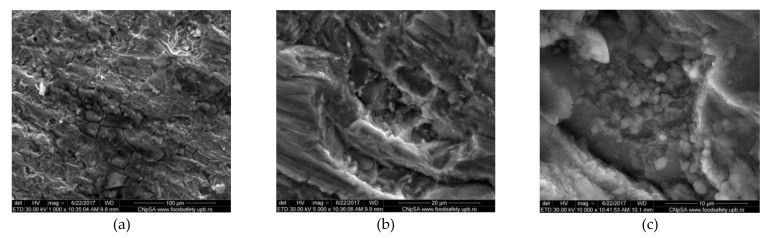

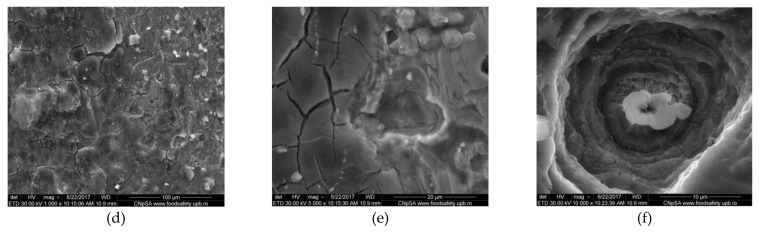
SEM micrographs (ETD images) of steel sample 1 (**a**–**c**) and steel sample 2 (**d**–**f**) after removing corrosion products formed after exposure 166 days in Fomtec; magnification from ×1000 (**a**,**d**) to ×10,000 (**c**,**f**).

Figure 1 and Figure 2 illustrate the morphologies of the corroded 42.2 k steel samples with and without corrosion products after the 166 days of immersion in Fomtec P 6%. Figure 1 shows SEM micrographs (ETD and CBS images) of surfaces covered with the corrosion products together with the EDS spectrum of the corrosion products. SEM images indicate that the steel immersed in Fomtec over a long period of time was covered with a non-compact film having numerous breaks, which represent sites for the continuous penetration of the aggressive liquid towards the metallic substrate, the process responsible for severe corrosion over time. Figure 2 shows, for two corroded samples, a non-regular surface visible after the corrosion products’ removal, suggesting the destructive attack occurred in the carbon steel bulk. At every magnification, the cracks, grooves and circular caverns in the sample mass were detected, all of which were the results of localized corrosion.

The composition of the corrosion product layer imparts certain protective abilities and strongly influences the subsequent corrosion behavior of the steel. In the literature of corrosivity of firefighting foams containing proteins the information about the chemical nature of corrosion products formed onto carbon steel surface is still scarce. It was found the akaganeite (β-FeOOH) layer as belt-like shaped crystallites for mild steel immersed in chloride containing aqueous environments [51,52]. Forms of other ferric oxyhydroxides, such as reddish-brown lepidocrocite (γ-FeOOH) and goethite (α-FeOOH fine crystallites with fibrous structure) were also reported as the main phases of corrosion layers together with FeOCl compound, and amorphous rust [53]. On contrary, an abundant literature has been published on corrosion of stainless steel in protein-containing media [54,55,56,57,58] whereas much less work has been dedicated to pure Fe [55,59,60]. For ferrous material surfaces, it is unanimously accepted that both physisorption and irreversible chemisorption of proteins may be possible and iron oxides formed on the surface will facilitate protein aggregation around the defect sites. Alternatively, it is possible for the protein to act as catalysts oxidizing the metal by their internal disulfate groups. Disulfide bonds in the new protein-metal complex compound may be subsequently reformed by oxidation with oxygen. This would also be the reason why contradictory findings are reported in the literature related to increasing or decreasing of corrosion attack in environments with protein content.

We have compared in our corrosion studies the chemical nature of the corroded products presents onto the surface to the bulk composition of steel. Thus, we can see the changes in chemical composition. Table 2 summarizes EDS data obtained for corrosion product formed on steel surface. Expectedly, we found a decrease in iron content in corrosion product layer (remained 5.65 wt% compared to about 99% in mild steel) and its significant enrichments in oxygen, carbon, nitrogen and chlorine, i.e., non-metallic elements as components of the oxide/hydroxide compounds or salts. Moreover, a preliminary X-ray diffraction analysis indicated that the corrosion products are mostly non-crystalline.

#### 3.1.2. Corrosion of Al Alloy (AlSiCuMg Type)

Only preliminary weight-loss experiments were performed regarding the gravimetric determination of corrosion rates for this cast Al alloy. We have tested three identical samples of Al alloy immersed 7 or 14 days in Fomtec and Profoam concentrates. Unfortunately, the obtained data of gravimetric index (*kg*) and penetration rate index *CR* (taking density value of alloy as 2.77 g/cm^3^) in both protein concentrates were somewhat irrelevant and uncertain, in the sense that their weight-loss values were very different for each sample under identical conditions of immersion. Moreover, in many cases of profiled coupons, the removal of corrosion product by simple cleaning, brushing and washing (including rinsing with acetone) resulted in weight gain instead of weight loss. An explanation may be the existence of native aluminum oxide, a strong adherence of corrosion products and a pore filling process. We mention that the native aluminum oxide was not intentionally removed in the sample preparation to simulate the conditions in practice.

The weight losses reached almost constant values after 14 days of immersion of Al alloy, with calculated average corrosion rates of 0.914 ± 0.147 mdd (0.474 ± 0.076 mipy) in Fomtec and 2.107 ± 0.425 mdd (1.093 ± 0.214 mipy) in Profoam. However, it is worth mentioning that we also preliminarily weighed the samples after 7 days of immersion, and the determined average corrosion rates were 0.184 ± 0.027 mdd (0.095 ± 0.014 mipy) in Fomtec and 0.283 ± 0.050 mdd (0.147 ± 0.026 mipy) in Profoam. These surprising results can indicate increased corrosion only after long exposure time. We may suppose that the slow processes of chemical dissolution or chemical conversion of the native aluminum oxide film during the first few days are followed by intense attack that produces a non-protective layer of corrosion products. The corrosion of Al alloy may be considered to advance over time, and therefore, the values of gravimetric index and penetration rate indicate non-stable behavior of aluminum alloy in contact with the protein foam concentrate for a long time. Thus, we have demonstrated the aggressiveness of the foam concentrates on nozzles or Storz couplings made by cast aluminum alloy. Bertschy et al. [2] reported a corrosion rate of 1–3 mdd for 6061 aluminum alloy in contact with a light water concentrate, also proving that Al alloy has the best corrosion resistance to all other foam agents investigated. Sheffey and Wright [3] determined for 6061 Al alloy a penetration rate of 0.03–0.15 mipy which was much lower than for UNS G 10,100 steel or 304 stainless steel.

**Figure 3:**
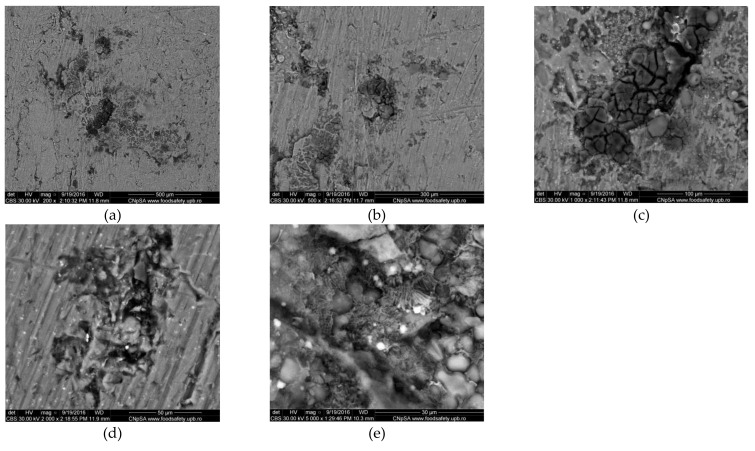
SEM micrographs (CBS images) of AlSiCuMg alloy corroded for 14 days in Fomtec after removing corrosion products; magnification (**a**) ×200; (**b**) ×500; (**c**) ×1000; (**d**) ×2000; (**e**) ×5000.

**Figure 4:**
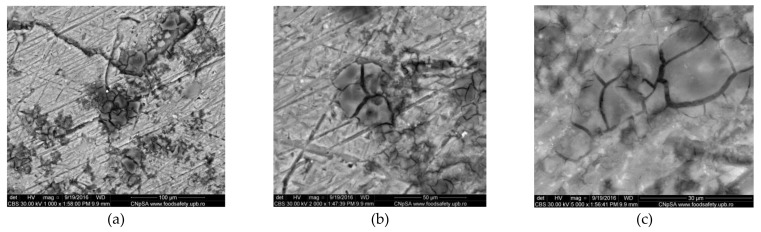
SEM micrographs (CBS images) of AlSiCuMg alloy corroded for 14 days in Profoam after removing corrosion products; magnification from (**a**) ×1000; (**b**) ×1000; (**c**) ×5000.

SEM images obtained for the initial aluminum alloy samples have illustrated interdendritic arrangement of silicon in the solid solution based on Al, and the existence of insoluble iron leading to acicular or polyhedral morphology. The quite uniform presence of Al, Si, Fe and Cu in alloy was identified in the EDS maps proving that the manufacturing company processed the Al alloy by appropriate heat treatments. Examples of the states of the cleaned surfaces after immersion of the Al alloy in Fomtec for 366 h and the alloy in Profoam for 350 h are shown, respectively, in Figure 3 and Figure 4, indicating that a part of the corrosion products remained as an adherent layer. Therefore, the morphology of corroded samples varied quite a lot based on the selection of the area for the microscopy, although the exposure conditions in the aggressive foam concentrate were the same. It can be clearly observed that large aggregates of corrosion products remained on some metal areas and their visible cracks. From the anticorrosion protection point of view, this is a drawback, meaning that the corrosion products deposited do not protect. In most cases, the Al alloy surface exposed in Profoam was much more corrosively attacked than in Fomtec, evidenced by more intense localized corrosion, deeper pits and caverns.

**Figure 5:**
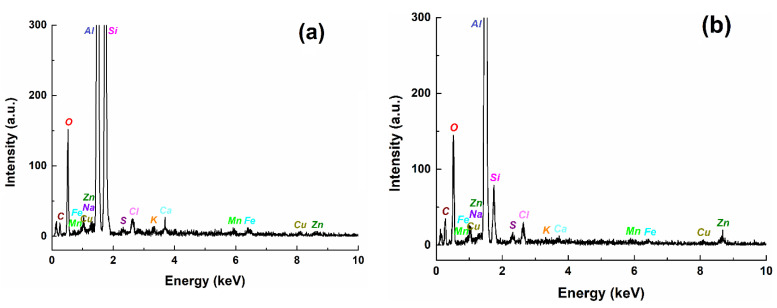
EDS spectra for corrosion products formed by corrosion of AlSiCuMg alloy for 14 days: in Fomtec (**a**) and in Profoam (**b**).

If the composition of the corrosion products (indicated in EDS spectra, Figure 5 and Table 3) is compared with the composition of Al alloy, the changes can be observed for all concentrations of the constituent elements. Thus, Al content decreased drastically from 77% to 35–40%, while silicon, which had a maximum of 13% in the alloy, had a variable concentration in the corrosion product—more than double the content (33%) after exposure to Fomtec and very low content (4%) after corrosion in Profoam. The contents of Cu, Fe and Mn decreased significantly. There are also elements such as oxygen (26–30%), carbon (21% for sample corroded in Profoam) and chlorine (around 1%) that were constituents of the corrosion products.

### 3.2. Studies of the Accelerated Corrosion Using Electrochemical Methods

The electrochemical corrosion tests were carried out to confirm that the weight loss data obtained are reliable. Plotting the potentiodynamic polarization curves and electrochemical impedance spectra represents a very useful procedure with which to evaluate the corrosion parameters within a short period of time [61]. Semi-logarithmic potentiodynamic polarization curves (Tafel plots) were recorded in a potential range around the free-corrosion potential (open-circuit potential OCP), generally by first polarizing the electrode 200–400 mV more cathodic and then 800–1000 mV more anodic. Values of corrosion potential and corrosion current were determined graphically from the intercept of the anodic and cathodic Tafel lines plotted from both branches of polarization curve. The corrosion current density I_corr_ (μA/cm^2^) can be converted to penetration corrosion rate *CR* (in mipy) by using Equation (1) based on Faraday’s law:

(1) $$CR=\;\frac{8760\times 10^{-6}\times 10^{3}}{26.8\times 2.54}\;.\;\frac{I_{corr}\;M}{n\;\;\rho }\;=\;0.12868\;\frac{I_{corr}\;M}{n\;\;\rho }$$

where *M* is the atomic weight of specimen (55.85 g/mol for Fe and 26.98 g/mol for Al), n is the number of electrons transferred in the corrosion reaction (*n* = 2 for Fe and *n* = 3 for Al) and ρ is the density (g/cm^3^) of the metal specimen. Remember that our working electrode had a 1 cm^2^ surface area exposed.

**Figure 6:**
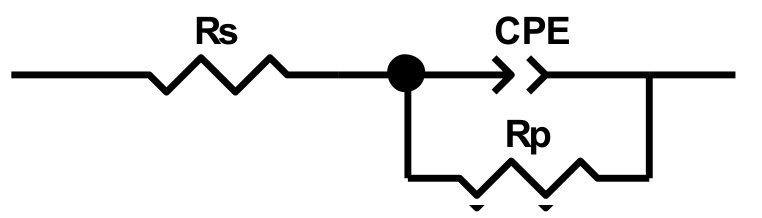
The equivalent electrical circuit proposed for modeling corrosion processes, with polarization resistance Rp as Faradaic impedance.

The Nyquist diagrams (graphical representation of imaginary impedance vs. real impedance) and Bode diagrams (graphical representation of phase angle vs. frequency) were plotted at OCP and various anodic potentials. The Nyquist semicircle diameter represents polarization resistance Rp which is inversely proportional to corrosion current. Applied anodic polarization was considered sufficient to illustrate the changes of corrosion behavior (corrosion current) that the metallic electrode underwent each time in fresh foam concentrate. Checking the validity of the electrochemical impedance spectroscopy data was performed by modeling with Randles type equivalent electrical circuit (Figure 6). In this circuit, *Rs* represents the ohmic resistance (uncompensated resistance) of the solution between the working electrode and auxiliary electrode. *Rs* was in series with a parallel circuit of an interface capacity and a resistance (the polarization resistance *Rp*) corresponding to the charge transfer in the corrosion process. Due to the presence of surface roughness and non-homogeneities of the coupon surfaces, a constant phase element CPE was used instead of a pure capacitor. CPE has the components *CPE-T* (capacitive element) and *CPE-p* (exponent representing the deviation from an ideal capacitor, for which *CPE-p* equals 1).

#### 3.2.1. Polarization Curves and EIS Spectra of Carbon Steel

**Figure 7:**
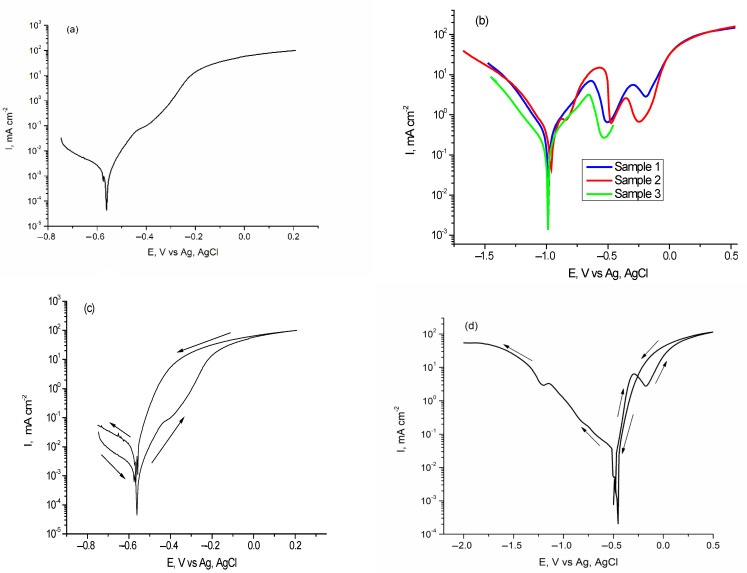
Potentiodynamic polarization of 42.2 k steel samples immersed at room temperature in Fomtec (**a**) and in Profoam (**b**); cyclic polarization of steel in Fomtec (**c**,**d**).

Flat steel plates were prepared from the walls, bottoms or lids of containers where Fomtec and Profoam foam concentrates are stored. Figure 7 presents potentiodynamic polarization curves for tested carbon steel samples in both investigated concentrates from which corrosion potential (*Ecorr*) and corrosion current density (*Icorr*) were determined graphically for each coupon.

By scanning the electrode potential in the cathodic direction, the curves reproduced themselves quite accurately and correspond to the electrochemical reduction of the atmospheric O_2_ molecules dissolved in the liquid. However, on the anodic branches, which correspond to the steel corrosion processes, there are clearly differences in the behavior of the samples. In steel/Fomtec system an active corrosion zone is observed in the beginning of curve (Figure 7a) due to the acceleration of iron dissolution caused by the strong action of proteins and inorganic compounds contained. This is followed by a fairly narrow range of constant current around the potential of −400 mV, after which the current increases again until it reaches a stationary state of constant and significant corrosion. Therefore, steel passivation was not observed in this system. In Profoam (Figure 7b), after the active corrosion zone, it followed a steady state characterized by sinusoidal variations of the current assigned to periodic processes of formation/detachment of adsorbed layers on the electrode. In this potential range the current has a decrease of at least an order of magnitude (a sinusoid minimum of *ca* 0.3 mA/cm^2^ can be seen on logarithmic scale), indicating temporary passivation due to the deposition of corrosion products. The process was clearly reproducible for all samples immersed in Profoam. The final anodic polarization led to a current that was almost constant, limiting the current characteristics of the systems that have a mechanism of diffusional control corrosion. Curves recorded by cyclic polarization (Figure 7c,d) show that when returning the potential scan, no other processes were observed along with the corrosion (in anodic branch) and oxygen reduction (in cathodic branch). Smaller hysteresis area during descending the anodic current indicates a lower pitting attack. Table 4 lists the corrosion parameters determined from Tafel polarization curves. Corrosion rate, calculated as penetration rate (mipy) using Equation (1), is included. Although penetration was of an order of magnitude larger than the CR values determined by the gravimetric method (Table 1), these data obtained by accelerated method confirm the greater aggressiveness of the Profoam concentrate on carbon steel in the short time (half an hour) in which the electrochemical measurements were performed.

**Figure 8:**
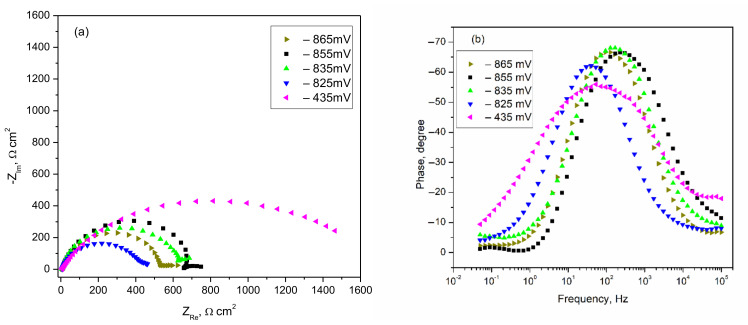
Nyquist spectra (**a**) and Bode phase angle spectra (**b**) for steel in Profoam at various electrode potentials starting from −865 mV (OCP).

Figure 8 shows a series of electrochemical impedance spectra recorded for 42.2 k steel samples in Profoam foam concentrate. Starting from the OCP, which has the value −865 mV vs./AgCl, various EIS spectra for more and more anodic polarization up to overvoltage of 430 mV were performed. For each Nyquist curve in Figure 8a, we observed an imperfect depressed capacitive semicircle which has at its end (at low frequencies) a linear portion, a “tail,” which can be attributed to the formation of a film on the electrode surface. A horizontal line would indicate an unstable film, suggesting a layer of corrosion products that periodically come off or are soluble in the electrolyte (unstable passivation). At high anodic polarization, the semicircles tend to have a worm-like, twisted inward shape, meaning that the corrosion products formed are more strongly adsorbed. However, the main feature of semicircle diameter (i.e., polarization resistance *R_p_*) is the existence of two trends of variation with anodic polarization: (i) at potentials between −865 and −835 mV a fluctuant value of the diameter (500–700 Ωcm^2^) is observed, proving an active corrosion; (ii) in the range of large polarization from −825 mV to −435 mV, the sudden growth of the diameter suggests a substantially diminished corrosion due to passivation. The phase angle–frequency dependence showed by Bode diagrams exhibits each time a single maximum phase angle. Almost constant value around −70° at low anodic polarization (including −835 mV) indicates strong barrier properties of the surface oxide as semiconductor corrosion products deposited on the steel’s surface. Recall that a maximum angle of –90° corresponds to an ideal electrical insulator. One might think that the film is always quite compact, but at the final high anodic polarizations of −825 and −435 mV, the maximum angle decreased gradually to −55°, which suggests less electric insulation, confirming an instable non-protecting layer.

The numerical results obtained by best fitting corrosion data on the presented model are listed in Table 5 and indicate plausible values for all cases of anodic polarization. The most reliable results are for the ohmic resistance of solution *Rs*, where the values are reproducible—most being in narrow range of 5.9–6.8 Ωcm^2^. The *CPE–T* parameter indicating the capacity of the electrical double layer has values 23–200 μF/cm^2^ which correspond to the usual range for double layer of metals in aqueous solutions. However, the steel/Profoam electrolyte interface behaved almost non-ideally, with the exponent *CPE–p* of 0.66–0.89 (less than 1). Although the evolution of the polarization resistance *Rp* during anodic polarization had some fluctuations, an active corrosion zone for polarization up to −825 mV can be suggested, followed by passivation illustrated by much higher values of Rp.

Our experimental results are consistent with the literature illustrating how the presence of proteins accelerates or decreases the steel corrosion. It has been shown by drawing cyclic polarization curves [54] that proteins such as transferrin, fibrinogen, gamma-globulin and albumin (all from bovine serum) increase in that order the corrosion of both 304 and 316L stainless steels in Hank human simulated fluid. Increases in anodic current density and intensification of the release of metal ions were observed. Acting as complexing agents, the proteins stimulate the metal dissolution and suppress the formation of a protective oxide layer. Corrosion for six different types of stainless steels in a cellular fluid, which had a same protein composition as the skeletal muscle, was investigated by Rojas and Lago [55]. Anodic polarization plots and cyclic polarization curves suggested a chemosorbed layer on the stainless steel consisted of a mixture of metal oxides and proteins. It seems to be a more unstable passive layer than that formed in protein-free solutions; the pitting corrosion was also observed. Tafel curves revealed a diffusion of protein control process of the corrosion mechanism. Atapour et al. [58] investigated corrosion of austenitic (AISI 316 L), ferritic (AISI 430) and lean duplex (LDX 2101) stainless steels used in the dairy industry. Potentiodynamic polarization curves and EIS spectra were recorded for simulated milk and whey protein isolate. All steel grades revealed low corrosion rates in the whey protein solution without any sign of active/metastable corrosion. A severe pitting corrosion was evident for 430 steel in simulated milk, whereas no pitting was taking place for either 316L or 2101 steel. The Nyquist diagram recorded by these authors clearly demonstrated the highest passivity for 2101 steel, followed by 316 L and 430 steel, in both food solutions. The maximum phase angle approaching −80° indicated that the barrier layer formed during corrosion hinders the migration of aggressive species, such as O^2−^ and Cl^−^, from the solution into the steel/solution interface. The influence of bovine serum albumin (BSA) and lysozyme on pure Fe behavior [59] has been studied in simulated body fluid (SBF). The authors observed intense corrosion explained by electrostatic interactions between different species of electrically charged oxide/hydroxide, newly formed on the electrode, and protein molecules existing in solution or adsorbed. However, it is worth mentioning that contradictory findings have been reported in the literature related to increasing or decreasing of corrosion in fluids containing proteins. For instance, Geringer et al. [56] recorded EIS spectra for 316L stainless steel in Ringer and NaCl solutions at different anodic polarizations and found that albumin at concentrations of up to 20 g/L positively influences (in the sense of diminished corrosion) the stability of steel. By adding the casein natural protein in HCl solution, other authors [62,63] found casein to inhibit the corrosion of both mild steel and 316L stainless steel.

#### 3.2.2. Polarization Curves and EIS Spectra of AlSiCuMg Alloy

**Figure 9:**
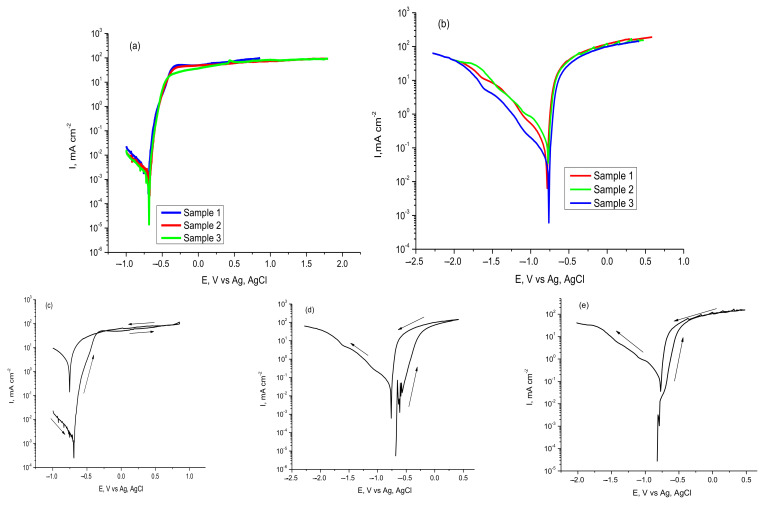
Comparative Tafel polarization curves for AlSiCuMg alloy in Fomtec (**a**) and in Profoam (**b**); cyclic polarization of Al alloy in Fomtec (**c**) and in Profoam (**d**,**e**).

We showed that aluminum alloy samples were taken by cutting profiled coupons from different nozzles used for spraying the foam or Storz couplings for hoses. Potentiodynamic polarization curves recorded in both investigated foam concentrates are presented in Figure 9 and were processed by the usual graphical procedure to determine the corrosion potential (*Ecorr*) and corrosion current density (*Icorr*).

In Figure 9, the branches of polarization curves at the most negative potentials (left) correspond to the cathodic reduction of dissolved oxygen. The oxygen molecules existed in solution because no deaeration (not bubbling with argon or pure nitrogen) was performed. It is possible to have a certain amount of absorbed reactive atomic hydrogen produced by the humidity in the environment [44,45], as discussed in the Introduction.

The anodic potential scanning (right branch) illustrates the processes during Al alloy corrosion in foam concentrate, with an active corrosion region starting from the open-circuit potential. The shapes are almost identical for both concentrates (Figure 9a,b) with the current density sharply increasing and then showing a current limitation within a potential range from −300 to +1800 mV. The active–passive transition was not clearly observed, although for Profoam we recorded small oscillations of anodic current in the active corrosion region between −600 and −500 mV. The wide region of limited current at high anodic potentials corresponds to a steady state in which corrosion occurred intensely and continuously, but the current value was kept constant due to a stationarity created between the amount of newly formed corrosion products which remained as the adsorbed layer and non-adherent products which fell (or dissolve) into solution. Scanning back the potential in cyclic polarization (Figure 9c–e) showed no other processes along with the corrosion of Al alloy (in anodic branch) and oxygen reduction (in cathodic branch).

Values of corrosion parameters determined for Al alloy immersed in foam concentrates are summarized in Table 6. It can be observed that Profoam is more aggressive against an aluminum alloy because the corrosion rate (expressed as *Icorr* and *CR*) is almost four times higher than that of Fomtec. The greater aggressiveness of Profoam also corresponds to what we found in the potentiodynamic polarization experiments for steel/foam concentrates systems. However, on the whole, the corrosive attacks of both foam agents were an order of magnitude lower for Al alloy than for 42.2 k steel.

Our results can be interpreted according to various investigations reported in literature. The effect of proteins on the corrosion of pure Al powder has been studied by Clark et al. [64], who found that the corrosion process in Ringer solution or NaCl solution is not significantly influenced by adding serum albumin or fibrinogen. In our experiments, the presence of proteins in the foam concentrate would have been responsible for the corrosion of Cu (a component with a high concentration in our AlSiCuMg alloy) rather than of the aluminum component, which is a highly passivated metal. Therefore, the attack probably became more intense than for pure aluminum metal, with the formation of globular corrosion products [65]. In our study of Al alloy behavior, its corrosion may be also correlated with the existence of few particles of intermetallic compounds (IM). IM particles are generally considered to be sites of localized corrosion initiation, due to the appearance of galvanic couples between the IM and the metal oxides that surround them. For example, it has been suggested [66] that the (Al, Cu)_x_(Fe, Mn)_y_Si intermetallic compound appears in the AA2024-T3 alloy, together with other modified forms of intermetallic compounds of the types Al8Fe2Si or Al10Fe2Si.

**Figure 10:**
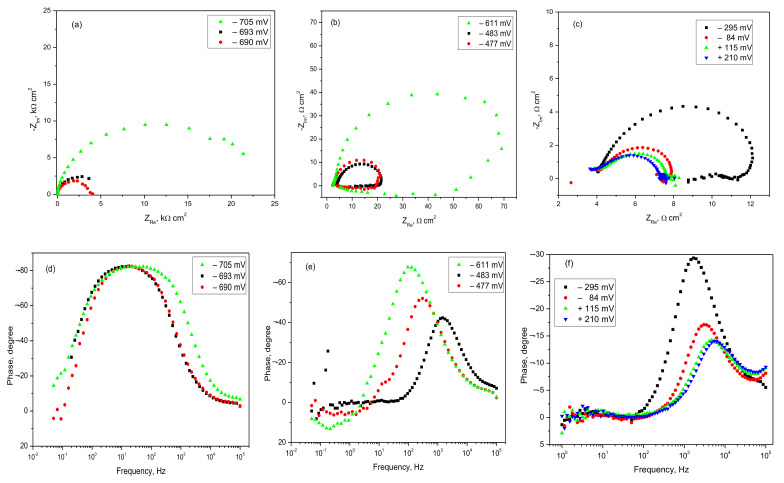
Nyquist spectra (**a**–**c**) and Bode phase angle spectra (**d**–**f**) for AlSiCuMg alloy in Profoam at various electrode potentials starting from −705 mV (OCP).

Figure 10 presents a series of EIS spectra obtained by gradual anodic polarization of Al alloy in Profoam. Simple capacitive Nyquist semicircles were observed for the first three polarizations near OCP (Figure 10a). At stationary potential the diameter of semicircle is around 25,000 Ωcm^2^ and then decreases to a few thousand Ωcm^2^, indicating an extension of the induction time for the active corrosion zone. Nyquist semicircles at higher anodic polarization levels (Figure 10b,c) have a worm shape, characteristic of corrosion with the formation of insoluble products that are adsorbed on the electrode. Their diameters have values that still decrease gradually with anodic overpotential, confirming the more and more intense corrosion of the Al alloy. In the Bode diagrams of the phase angle vs. frequency, a single maximum phase angle is shown each time. Figure 10d exhibits a wide frequency range for a Bode curve, in which the maximum phase angle of −80° is maintained (including −690 mV polarization), which indicates strong electrical insulation of the surface due to native aluminum oxide which has not yet been corroded. This is in agreement with weight-loss data. Figure 10e,f shows a drastic decline in the maximum phase angle, from −70° to −13°, confirming a continuous corrosion process and the formation of a porous layer with less and less protection against corrosion.

The quantitative results of the best spectra fitting are provided in Table 7. Although constant values of the ohmic resistance of the solution (3-4 Ωcm^2^) were obtained, the Rs is a little smaller than that obtained for 42.2 k steel corrosion in the same liquid concentrate. Plausible values of the capacity of the electrochemical double layer (*CPE-T* value of 11–125 μF/cm^2^) were calculated, which are usual for a metal/aqueous solution interface. The values of the *CPE-p* exponent are in the range 0.92–1, which confirms that the Al alloy behaves almost as an ideal double-layer capacitor. In the final column, *Rp* polarization resistance decreases continuously with more anodic polarization, confirming the continuous process of Al alloy corrosion shown by polarization curves (Figure 9). It should be noted that imposing maximum anodic overpotential of 1 V from the stationary potential (at +210 mV) led to an EIS spectrum that could not be modeled correctly by the proposed equivalent circuit. In said case, more complex circuits are needed as models—for example, with two parallel circuits connected in series with *Rs*.

## 4. Conclusions

Weight-loss measurements of mild steel and aluminum alloy (AlSiCuMg) corroded in Fomtec P 6% and 6% P Profoam 806 protein-based foam concentrates showed that Al alloy experienced a lower weight-loss rate than the carbon steel.

However, the general tendency of both materials is to reach the largest corrosion rate in the first period of exposure, followed by a stationary state for prolonged immersion, during which almost constant corrosion rates were recorded.

SEM images exhibited the formation of layers containing corrosion products and localized corrosion occurrence. EDS elemental analyses showed a decrease in metal (iron, aluminum) content in the corrosion product layer (compared to about 99% in mild steel and 77% in Al alloy) and significant enrichment in oxygen, silicon, carbon, nitrogen and chlorine—i.e., non-metallic elements as components of the oxide/hydroxide compounds or salts.

The corrosion rates calculated from corrosion current density using the potentiodynamic polarization are in good agreement with those obtained from weight-loss measurements, although they are an order of magnitude higher. No obvious passive region was recorded, except for some current oscillations within the anodic branch observed for both steel and Al alloy in Profoam, which confirms once again the greater aggressiveness of the Profoam than Fomtec. Impedance spectra showed each time a single capacitive semicircle on the Nyquist curve and a single maximum phase angle on the Bode diagram.

Calculated data for the best fitted elements of the simulated equivalent circuit revealed plausible values for solution resistance and capacity of the electrical double layer. Values of polarization resistance for steel corrosion had small fluctuations, which may have been due to the fact that iron as a steel alloying element can form some stable compounds in the presence of hydrolyzed proteins; clear passivation occurred only at high anodic polarization. Polarization resistance for Al corrosion decreased continuously with more anodic polarization, confirming the continuous process of corrosion.

Regarding the action of proteins existing in the foam concentrates, they can promote complexation of metal (iron, aluminum) cations in the electrolyte, and therefore, uniform corrosion is accelerated by their diminished concentration. All reported results can be used to improve safety in firefighting services.

## Figures and Tables

**Table 1:** Corrosion rates expressed as gravimetric index (kg) and penetration rate for 42.2 k steel in the two foam concentrates.

Steel Sample	*kg* (mdd) after Different Immersion Periods of Time. in Brackets: Penetration Rate (mipy)				
	29 Days	63 Days	81 Days	232 Days	348 Days
Fomtec P 6% concentrate					
1	2.551 (0.466)	3.233 (0.591)	3.278 (0.599)	2.990 (0.547)	2.962 (0.543)
2	2.654 (0.484)	3.190 (0.583)	3.204 (0.587)	3.396 (0.622)	3.672 (0.673)
3	3.552 (0.650)	4.258 (0.779)	4.236 (0.776)	5.568 (1.016)	5.594 (1.020)
Average	2.919 ± 0.550 (0.533 ± 0.060)	3.560 ± 0.604 (0.651 ± 0.111)	3.573 ± 0.576 (0.654 ± 0.106)	3.985 ± 1.386 (0.728 ± 0.252)	4.076 ± 1.362 (0.745 ± 0.247)
**6% P Profoam 806 concentrate**					
4	7.174 (1.311)	9.602 (1.756)	11.424 (2.087)	8.693 and 7.226 * (1.589 and 1.311 *)	10.205 (1.866)
5	8.798 (1.610)	13.994 (2.559)	14.304 (2.614)	11.194 and 9.523 * (2.047 and 1.740 *)	12.180 (2.228)
6	7.411 (1.354)	9.890 (1.807)	11.513 (2.106)	9.103 and 7.810 * (1.665 and 1.429 *)	10.586 (1.937)
Average	7.794 ± 0.877 (1.425 ± 0.162)	11.162 ± 2.457 (2.041 ± 0.446)	12.414 ± 1.482 (2.269 ± 0.299)	9.663 ± 1.341 (1.767 ± 0.245)	10.990 ± 1.012 (2.010 ± 0.192)

* These additional results are differential corrosion rates calculated from experimental data within the period of 81–232 days.

**Table 2:** The main elements detected by EDS in the corrosion product onto steel surface after 166-day immersion in Fomtec P 6%.

Chemical Composition	Elements									
	Fe	Mn	S	Ca	Na	K	C	O	N	Cl
wt%	5.65	1.05	0.67	0.48	5.48	0.20	36.98	34.69	10.72	4.08
at%	1.55	0.25	0.32	0.18	3.65	0.08	47.22	33.25	11.74	1.76

**Table 3:** The main elements detected by EDS in the corrosion products on the Al alloy surface after immersion for 14 days in Fomtec or Profoam.

Chem. Compos.	Elements												
	Al	Si	Cu	Fe	Mn	Zn	Ca	Na	K	S	C	O	Cl
Corroded Al alloy in Fomtec													
wt%	35.25	33.92	0.35	0.54	0.39	0.46	0.51	0.49	0.26	0.34	-	26.20	1.28
at%	30.61	28.29	0.13	0.23	0.17	0.17	0.30	0.50	0.16	0.25	-	38.37	0.85
Corroded Al alloy in Profoam													
wt%	40.90	4.40	0.47	0.33	0.48	1.28	-	0.33	-	0.47	20.95	29.42	0.98
at%	28.31	2.93	0.14	0.11	0.16	0.37	-	0.27	-	0.28	32.58	34.34	0.52

**Table 4:** Corrosion parameters determined from potentiodynamic polarization curves of steel in foam concentrates.

Corrosion System: Steel/Foam Agent	*OCP*, mV	*Ecorr* vs. Ag/AgCl, mV	*Icorr*, μA/cm^2^	*CR*, mipy
Sample 1 in Fomtec	−526	−561	43.5	19.887
Sample 2 in Fomtec (cyclic)	−538	−566	48.2	22.036
Sample 3 in Fomtec (cyclic)	−501	−538	64.6	29.533
Average:			52.1 ± 10.98	23.818 ± 5.06
Sample 1 in Profoam	−862	−984	194	88.690
Sample 2 in Profoam	−887	−929	238	108.806
Sample 3 in Profoam	−865	−987	172	78.633
Average:			201.3 ± 33.60	92.043 ± 15.36

**Table 5:** Values of equivalent circuit parameters for corrosion of 42.2 k steel in Profoam concentrate.

Anodic Polarization, mV	*Rs*, Ωcm^2^	*CPE−T*, μF/cm^2^	*CPE1−p* Exponent	*Rp*, Ωcm^2^
−865	7.2	48.54	0.875	550
−855	6.8	23.33	0.862	699
−845	6.4	29.83	0.890	526
−835	6.4	126.49	0.746	605
−825	7.7	177.33	0.825	439
−795	5.9	41.29	0.852	1180
−435	5.1	204.44	0.664	1558

**Table 6:** Corrosion parameters determined from potentiodynamic polarization curves of Al alloy in foam concentrates.

Corrosion System: Al Alloy/Foam Agent	*OCP*, mV	*Ecorr* vs. Ag/AgCl, mV	*Icorr*, μA/cm^2^	*CR*, Mipy
Sample 1 in Fomtec	−705	−695	1.45	0.606
Sample 2 in Fomtec	−680	−677	1.93	0.806
Sample 3 in Fomtec	−676	−677	1.32	0.551
Average:			1.57 ± 0.321	0.654 ± 0.134
Sample 1 in Profoam	−745	−747	10.0	4.178
Sample 2 in Profoam	−729	−713	7.1	2.966
Sample 3 in Profoam	−776	−781	9.1	3.802
Sample 4 in Profoam	−705	−702	6.3	2.632
Average:			8.12 ± 1.717	3.394 ± 0.718

**Table 7:** Values of equivalent circuit parameters for corrosion of Al alloy in Profoam concentrate.

Anodic Polarization, mV	*Rs*, Ωcm^2^	*CPE-T*, μF/cm^2^	*CPE-p*	*Rp*, Ωcm^2^
−705	3.3	111.98	0.939	25352
−693	3.2	97.01	0.948	5933
−690	4.4	28.60	0.926	2831
−611	3.0	125.47	0.926	78
−483	4.1	10.95	1.010	17
−477	2.8	99.39	0.938	16
−295	4.2	13.58	1.018	7.4
−84	4.2	13.06	1.016	3.5
+115	4.2	24.50	0.920	3.2

## Data Availability

The data presented in this paper are available on request from the corresponding author.
